# Herbivory rate is elevated but orb-weaver spider growth unaffected by artificial light at night in subtropical forest

**DOI:** 10.1007/s00114-025-02031-w

**Published:** 2025-10-14

**Authors:** Yirong Guo, Yiu Siu, John A. Allcock, Caroline Dingle, Louise A. Ashton, Timothy C. Bonebrake

**Affiliations:** 1 School of Biological Sciences, The University of Hong Kong, Hong Kong, China; 2 Department of Biology, Capilano University, North Vancouver, British Columbia Canada

**Keywords:** Arachnology, Ecological light pollution, Plant-herbivore, Predator–prey, Species interactions, Trophic interactions

## Abstract

Artificial light at night (ALAN) is a growing threat to biodiversity and ecosystems globally. However, a limited number of studies have focused on the effect of ALAN in the tropical or subtropical regions, and the impact of ALAN on species interactions and ecological processes is particularly understudied. We introduced ALAN into light-naïve forest plots to examine whether ALAN affects herbivory rate and alters the growth and abundance of *Nephila pilipes*, a common orb-weaver spider. We found illuminated plots had a higher herbivory rate than control plots in the early wet season. The growth rate and abundance of the spider species, however, were not affected by ALAN. Our results indicate that ecological processes in tropical ecosystems, such as herbivory, are potentially sensitive to ALAN. Additionally, this study highlights the importance of a more mechanistic understanding of the sensitivities of tropical species interactions to ALAN and the possible complications caused by environmental variation.

## Introduction

Increasing artificial light at night (ALAN) is a major anthropogenic threat to global biodiversity (Hölker et al. 2010). ALAN has widespread effects on the physiology and life history traits of species (Longcore and Rich 2004; Sanders et al. 2021). Studies have increasingly reported that ALAN alters community structure and composition (Davies et al. 2012, 2017; Spoelstra et al. 2023) and affects interactions between trophic levels via specific interactions, such as predation, herbivory, and host-parasitoid relationships (Grubisic and van Grunsven 2021). The introduction of ALAN is likely to further contribute to large-scale change in ecosystem stability and functions (Cesarz et al. 2023). This effect of ALAN on species interactions and ecosystems is further aggravated by the recent global trend of replacing traditional outdoor lighting with LED bulbs (Davies et al. 2017), since LED light is reported to generate a greater perceptive disparity across different taxonomic groups in ecosystems than narrow-spectrum lighting systems (Davies et al. 2013).

Ecological light pollution can affect long-evolved predator–prey interactions in multiple ways. Several studies suggest that ALAN leads to elevated predation rates. By altering predator avoidance behaviour and causing spatial disorientation in some moth species, ALAN can increase predation risk (Acharya and Fenton 1999). ALAN can disrupt prey visual systems (Briolat et al. 2021) and impair cryptic or aposematic colouration, making prey more easily eaten by predators (Delhey and Peters 2017; Owens and Lewis 2018). Positive phototaxis of both arthropod prey and predators is frequently observed. Positive phototaxis may be even stronger and longer lasting for predators, resulting in greater predation pressure in light-polluted areas (Davies et al. 2012; McMunn et al. 2019). Some predators have also adopted mechanisms to exploit ALAN. Although net-building spiders do not primarily depend on visual cues for foraging, they have been reported to leverage ALAN-polluted environments to their advantage. For example, some orb-web spiders build webs near artificial lights to capture prey attracted by the lights (Heiling 1999; Willmott et al. 2019), which may partially explain the success of orb-web spiders in urban areas (Lowe et al. 2014). However, prey can also benefit from ALAN through increased vigilance (Zhang et al. 2020) or increased visibility of predators (Yuen and Bonebrake 2017).

Herbivory is another ecological interaction sensitive to ecological light pollution. Several studies have shown that herbivory tends to increase under ALAN (Mondy et al. 2021; Cieraad et al. 2023). Exposure of some insect species to ALAN can directly increase herbivory through effects on life history traits (Schroer et al. 2019). Conversely, exposure to ALAN can lead to enhanced mechanical toughness and increased defences of plants, which would reduce herbivory levels (Salgado-Luarte et al. 2023; Cao et al. 2024). Some plants exposed to ALAN exhibit biomass decline which leads to a bottom-up effect on the herbivore community (Sanders et al. 2015). Increased predator populations due to ALAN can also decrease the population size of herbivores and impede the herbivory process, exhibiting a cascading effect (McMunn et al. 2019). Overall, the mechanisms underlying how ALAN affects herbivory via interactions between multiple trophic levels remain unclear.

Recent studies have employed experimental systems to introduce ALAN into light-naïve ecosystems, allowing for the investigation of the immediate response of ecosystems to light pollution. Deitsch and Kaiser (2023) discovered that a moderate level of ALAN (about 15 lx) increased the abundance of arthropod predators and parasitoids, increasing top-down control of caterpillars. McMunn et al. (2019) observed a rise in herbivory and predation rates following the introduction of ALAN to a grassland ecosystem. Given the effects observed in these studies from temperate ecosystems, tropical and subtropical ecosystems may be particularly vulnerable to ALAN effects due to complex trophic interactions – introducing ALAN experimentally could yield novel insights into light pollution impacts on these diverse ecological communities.

**Fig. 1:**
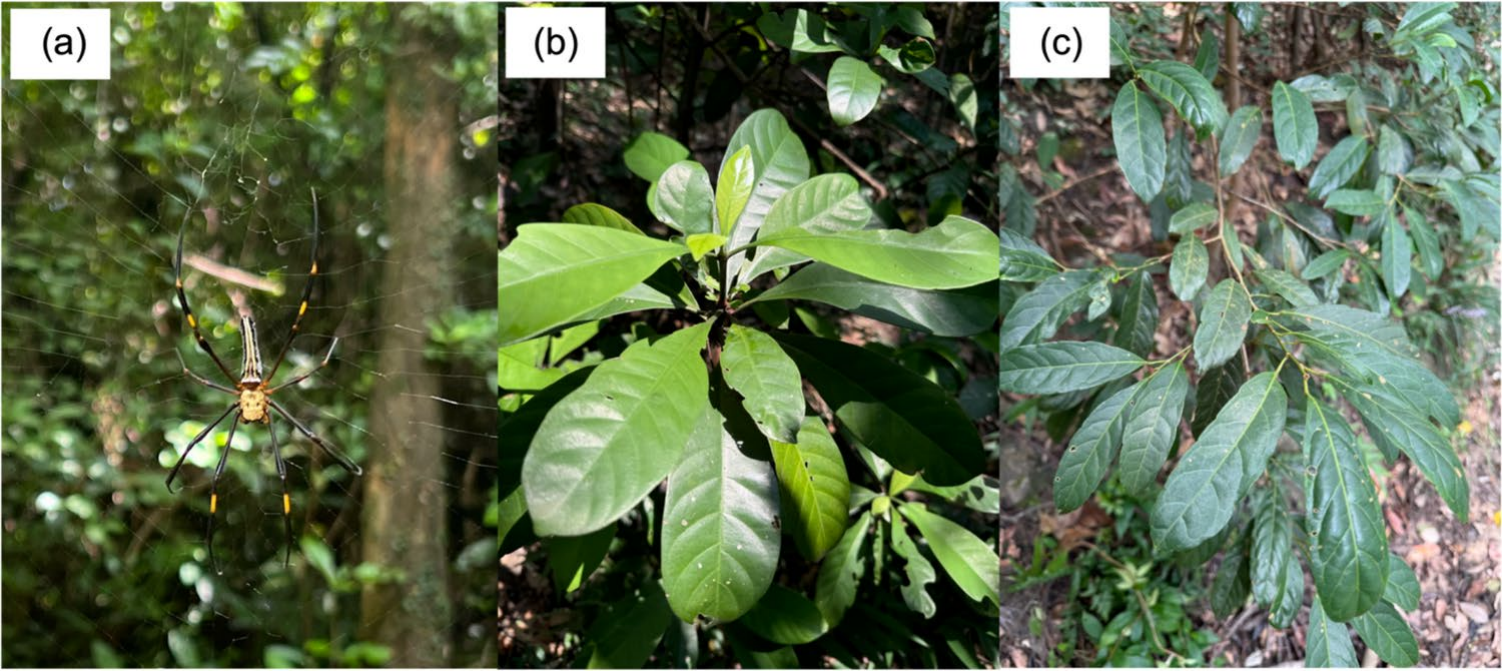
Images of *Nephila pilipes* and the two focal plant species of this study. (**a**) *N. pilipes* is a common spider species in the secondary forests of Hong Kong and can be found throughout the wet season. The conspicuous yellow pattern is critical for prey attraction; (**b**) *P. asiatica* (**c**) and *A. dioica* are prevalent understory plant species in the secondary forests of Hong Kong

We investigated predator (spider) and prey (as measured by herbivory rates) responses to ALAN through the experimental introduction of light in the subtropical forest ecosystems of Hong Kong. The spider species in our study, *Nephila pilipes* (Fabricius, 1793), a dominant species in Hong Kong secondary forest (Fig. 1a), is prominent between March and November (Yuen and Bonebrake 2017). This species exhibits sexual dimorphism, and female individuals are conspicuously larger than males (Kuntner et al. 2012). Typical females have yellowish-black prosoma, legs, and bright yellow ventral carapace. Additionally, a black morph of *N. pilipes* exists as part of an interbreeding population (Tso et al. 2002). These spiders hunt by luring prey with striking yellow patterns and UV-reflective markings on their bodies (Tso et al. 2004). Moths have been identified as a major nocturnal prey of the species (Chuang et al. 2007) and previous research has shown that ALAN reduces the moth prey interception rate of *N. pilipes*, potentially by enhancing the ‘visibility’ of the spider species to prey (Yuen and Bonebrake 2017). However, the counteraction between phototaxis of prey and decreased prey-interception has not been investigated. Following Yuen and Bonebrake (2017), we predicted that ALAN would decrease *N. pilipes* abundance, size, and growth rate. We also recorded herbivory rates from chewer herbivores in all experimental plots for two common understory plant species *Psychotria asiatica* L. (1759) and *Aporosa dioica* (Roxb.) Müll.Arg. (1866) (Fig. 1b-1c). The main herbivores of *P. asiatica* have not been reported in any study but based on our field observation and studies of other congeneric species (Novotny et al. 2002), the major herbivores are moth larvae. Although a similar level of herbivory was observed compared to *P. asiatica*, the specific herbivores of *A. dioica* remain unclear (though *A. dioica* are known targets of insect herbivory – Wen et al. 2006). As moths make up a major proportion of the nocturnal food source for *N. pilipes* and ALAN is reported to impair the moth interception of the spider species, we predicted that ALAN would reduce spider abundance and growth, which would thereby elevate herbivory rates as a result of insects (e.g. adult moths) having higher survival and reproductive rates in these areas, leading to more caterpillars and herbivory.

## Materials and methods

### Study site and light treatment

We introduced experimental lights into forests plots in Hong Kong to monitor the dynamics of *N. pilipes* growth and abundance, as well as herbivory. Hong Kong has a monsoon-dominated climate and shows a strong seasonal variation between the wet season (April to September) and the dry season (October to March) (Dudgeon and Corlett 2004). We conducted this study from the early to mid-wet season (April through June) in 2024, during which *N. pilipes* were mostly in their juvenile stage and their growth was more clearly detectable.

**Fig. 2:**
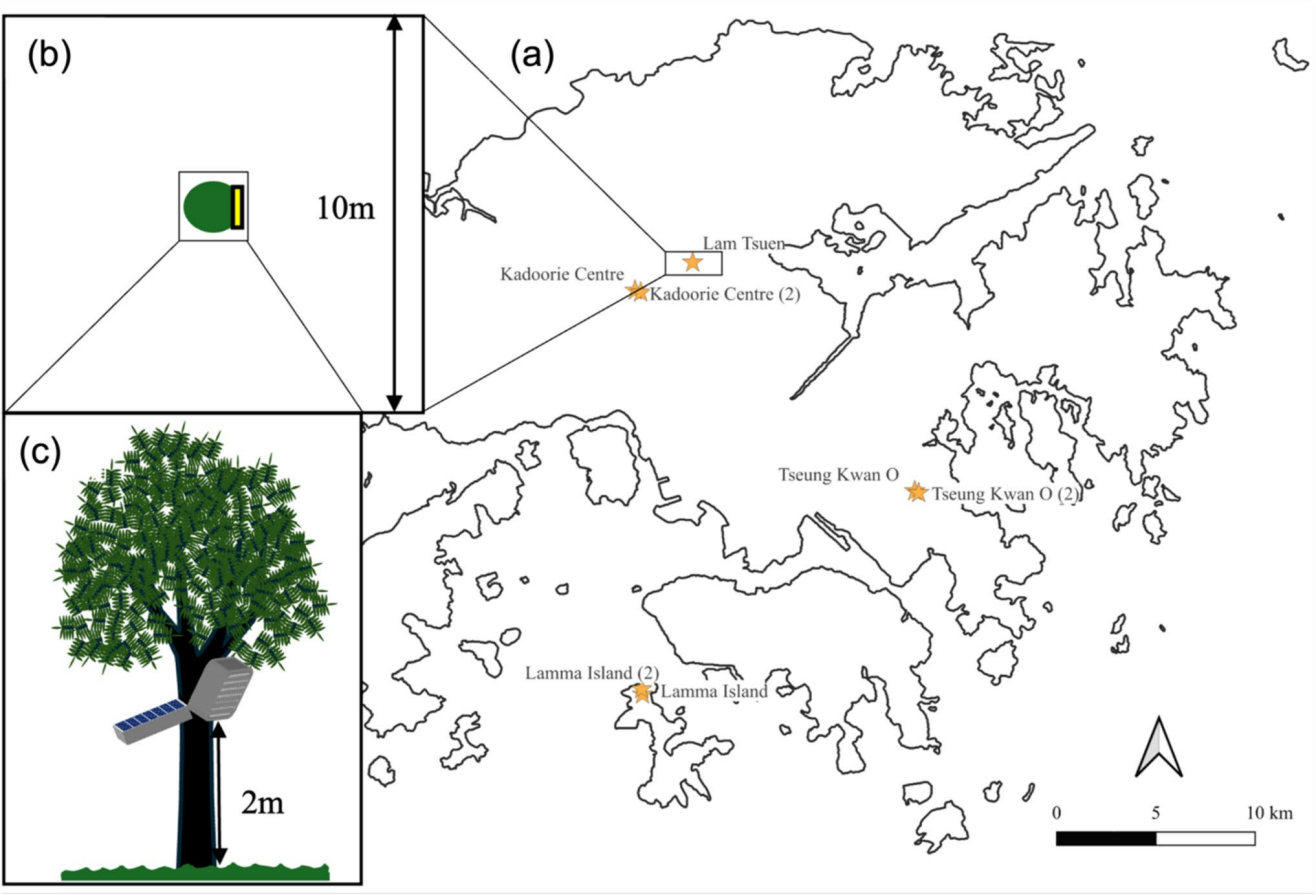
Contour map of study sites in Hong Kong, and schematic diagrams of plot setup and light treatment. (**a**) A total of 14 plots were set up in a pair design (7 pairs, marked as stars) across 4 sites in Hong Kong; (**b**) Each plot was established as a square of 100m^2^ with a light at the centre; (**c**) In each plot, the light and the solar panel were affixed to a tree trunk with a rope, 2 m above the ground, and the light was pointed downwards at roughly 45°. Experimental plots were illuminated, while the lights in control plots were not turned on

We conducted our experiments across four peri-urban sites in Hong Kong (Fig. 2a). We chose these sites due to the high abundance of *N. pilipes* observed in these sites during pre-study surveys. All sites were set to be at least 250 m away from the nearest residential area with artificial lights. Despite potential sky glow due to spillover of urban ALAN, peri-urban regions in Hong Kong have a much lower night sky brightness—up to 100 times less bright compared to urban areas (Pun and So 2012). Thus, we consider the chosen sites to be relatively naïve to artificial light at night. In total, we established 14 plots in a paired design (*n* = *7* in pairs, *n* = *2* for Lamma Island, *n* = *2* for Kadoorie Centre, *n* = *2* for Little Hawaii Falls, and *n* = *1* for Lam Tsuen) (Fig. 2a) during the late dry season (late March) and early wet season (early April) 2024. For each pair, an illuminated experimental plot was paired with an unilluminated control plot. Following Deitsch and Kaiser (2023), within each pair, individual plots were separated by at least 50 m to ensure that light spillover from the experimental plot did not affect the control plot. Also, pairs within sites were separated by at least 250 m to ensure the independence of each pair.

Every plot was set as a square of 100m^2^ in area and with a light at the centre (Fig. 2b). Boundaries were marked by flagging tape. Each experimental plot was illuminated by one solar-powered LED light (25W, Outdoor (IP67), cool-white, 6500 K, broad-spectrum) whose light beam could cover a circular area with a radius of four metres (i.e. illuminance level of 0 lx at a radius of four meters). The lamps used in our experiment share similar light properties with LED road lights globally (Jin et al. 2015). The same light was also set up in each control replicate but was not turned on. For all plots, the lights were affixed to a tree trunk with a rope two metres above the ground (distance measured by the iOS app *tape measure*) and pointed downwards at roughly 45° to the ground (Deitsch and Kaiser 2023) (Fig. 2c). The light treatment in our illuminated plots resulted in a nocturnal illuminance of 47 lx at a distance of 0.1 m, similar to the illuminance level measured from typical streetlights at 2 m above ground (Bennie et al. 2016).

Although LED is not as efficient as other UV-emitting illumination systems in attracting moths (Wakefield et al. 2016), which is a critical link between our hypotheses, we decided to adopt the LED treatment as this is a global trend to substitute conventional outdoor lighting with LED (Davies et al. 2017). Therefore, our experiment provides a realistic reference for future conservation and environmental management decisions. Additionally, the cool-white LED we adopted contains more blue light components than warm-white LEDs, making it more attractive to moths (Brehm et al. 2021).

### Spider abundance, size, and biomass index

From early to mid-wet season (mid-April to late June) 2024, every two weeks we sampled the size and abundance of spiders in plots. In every survey, we only sampled female *N. pilipes* in plots because the conspicuous size and colour pattern of female individuals enables easy identification. During the survey, most *N. pilipes* we encountered were juveniles, therefore data collected reflects how the growth of spiders was affected by the introduction of ALAN. In each survey, we counted and recorded the number of *N. pilipes* females in the plots. The size of *N. pilipes* individuals, defined as the sum of the length of cephalothorax and abdomen, was measured by taking a picture of the individual with a laminated scale bar. The size of the spider was then calculated from the image using *ImageJ* (Schneider et al. 2012) and the average size of spiders in each plot was calculated. We also used a biomass index of *N. pilipes* for each plot, defined as the sum of the sizes (length of cephalothorax and abdomen) of all female *N. pilipes* in each plot, as spider body size is a suitable indicator for biomass (Penell et al. 2018).

### Herbivory rates

From early to mid-wet season (mid-April to late June) 2024, the herbivory rate within each plot was sampled in three rounds, each sampling rounds lasted for 15 days. The focal species of our survey were *Psychotria asiatica* (Rubiaceae) and *Aporosa dioica* (Phyllanthaceae) (Fig. 2b; Fig. 2c). We chose these species because they are common species in the secondary forest of Hong Kong and were identified in all 14 plots. During each sampling, individuals of focal species within each plot were identified, and 10 fully expanded leaves were collected randomly from individual plants of each species (a total of 20 leaves for each plot for each survey). We collected fully expanded leaves to facilitate the estimation of herbivory. The light treatments were set up between the late dry season and early wet season. This timing ensured that most of the herbivory activity observed occurred during the experiment since insects or herbivores are generally less active during the dry season in Hong Kong. On the same day of leaf sampling, the area loss caused by chewing activity of herbivores and the potential original area of leaves were estimated using the *LeafByte* application (Getman-Pickering et al. 2020). Percentage of consumption area of every leaf was calculated using *LeafByte* with the equation.

$$\frac{\text{Area loss}}{\text{Potential Origianl Area}}*100\%$$

### Data analysis

In this study, we analysed how the abundance, size, and biomass index of *N. pilipes* and herbivory of the two focal plant species were affected by ALAN introduction and temporal variation. Our analysis was mainly conducted by constructing generalised linear mixed models (GLMM) and linear mixed models (LMM).

For the analysis of *N. pilipes* data, we focused on three response variables: average size, abundance, and biomass index. To test the prediction that introduction of ALAN would decrease the predation rate and thus impede the growth of *N. pilipes*, we constructed one GLMM with abundance as the response variable and two sets of LMMs with average size or biomass index as response variables. To account for temporal variation, we defined the ‘number of days’ for a specific survey as the time duration between the survey date and April 1 st, 2024. The global models of the three sets of models included two fixed effects: treatment (categorical: lit or unlit) and number of days. We included locations (LM1, LM2, KC1, KC2, LT1, TKO1, and TKO2) as random effects because the difference in field condition between pairs within the same site is comparable to the difference between pairs among different sites. For abundance, we fit the models with a Quasi-Poisson distribution due to the nature of count data and observed overdispersion in the data (Zuur et al. 2009).

To test the prediction that the introduction of ALAN would decrease the predation rates of *N. pilipes* and thereby indirectly increase herbivory, we constructed two sets of LMMs with herbivory percentages of *P. asiatica* and *A. dioica* as the response variable, respectively. We defined the ‘sampling round’ of each plant herbivory survey based on which round of survey (i.e. first, second, or third) was undertaken. The global model included treatment and sampling round as fixed effects. To account for the repeated measurements within each sampling location, we included location and sampling round nested within locations as a random effect. The random intercept of locations accounted for the variation among locations and the interaction between sampling round and location in the random effect structure models the within-site variability across sampling rounds, hence capturing the correlation of repeated measures within each site.

For all models, we followed the model selection procedure with a top-down strategy recommended by Diggle et al. (2002). As locations is the only random effect component and random intercept model is adopted in the analysis, we started with searching for the optimal fixed effect structure. If the interaction term or fixed effect was not significant in the GLMM or LMM, it was not included in the optimal fixed effect structure. This selection procedure was further verified by comparing the original model with the term-dropped model using ANOVA. The proportion variation explained by the random effect component was calculated as the difference between conditional and marginal ${R}^{2}$ (Nakagawa and Schielzeth 2013).

All statistical analysis and data visualisation in this study were conducted in R (v4.2.1; R Core Team 2022). GLMM analysis was performed using the MASS R package (v7.3.60; Venables and Ripley 2002). LMM analysis was conducted via the nlme package (v3.1.157; Pinheiro and Bates 2000; Pinheiro et al. 2022) and lme4 package (v1.1–34; Bates et al. 2015) in R.

## Results

### Effects of ALAN on spider abundance, size, and biomass index

**Fig. 3:**
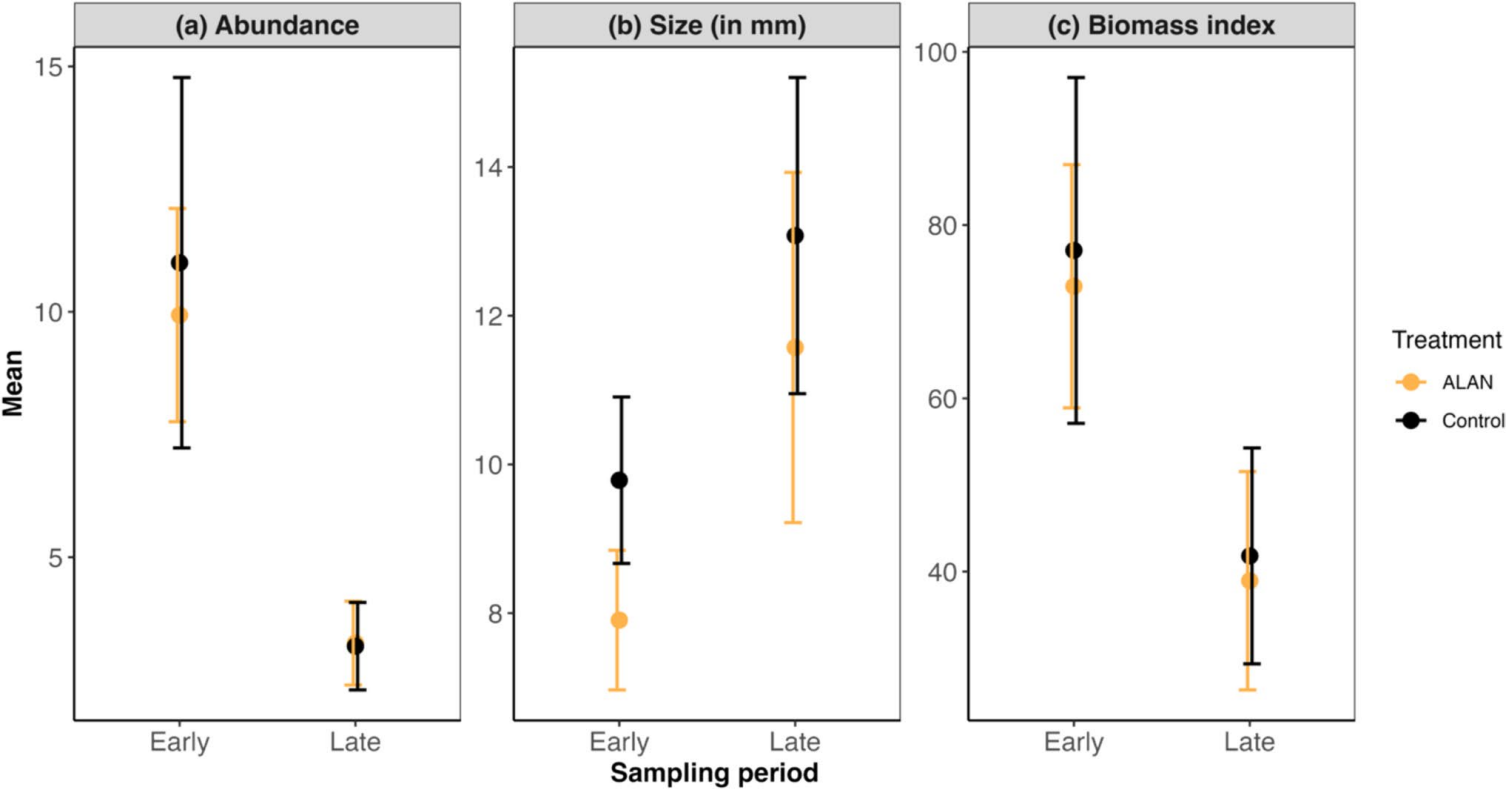
Mean *N. pilipes* abundance (**a**), size (**b**), and biomass index (**c**) across light treatment and sampling period. Error bars indicate mean ± standard error. Number of days was broken down into categorical variable for visualization, in which surveys conducted before 30th May 2024 were identified as early stage, otherwise as late. There are a total of 43 days and 31 days in the early and late stage of sampling, respectively

We did not find any significant impact of ALAN on the abundance, size, and biomass index of *N. pilipes*. Out of 417 female *N. pilipes* sampled in our experiment, 201 were found in illuminated experimental plots and 216 in unilluminated control plots. Mean (± 1 s.d.) *N. pilipes* size on experimental plots was 9.61 ± 6.51 mm and 11.43 ± 6.46 mm on control plots. The mean biomass index for *N. pilipes* on experimental plots was 55.94 ± 53.63 mm and 58.34 ± 66.19 mm on control plots. The best-performing models for abundance, size, and biomass index (Table 1) only indicated that number of days had a significant effect on *N. pilipes* (p < 0.001, p = 0.003, p = 0.001, respectively), reflecting growth over time (abundance decreased but size increased), while no significant impact of ALAN introduction was observed for any of the three response variables (Fig. 3).

**Table 1:** Parameter estimates (± s.e.), t-value, and P-value for each parameter included in the best-performing GLMM/LMM examining the effect of introduction of ALAN and number of days on *N. pilipes* abundance, size, and biomass at the 14 plots around Hong Kong. For each model, the marginal and conditional ${R}^{2}$ is presented to assess the contribution of random effect (location) to the sample variation

response variable	parameter	estimate ± s.e	t	p	marginal/conditional${R}^{2}$
abundance	intercept	2.65 ± 0.37	7.18	< 0.001	0.203/0.823
	number of days	–0.02 ± 0.00	–6.23	< 0.001	
size	intercept	4.96 ± 2.26	2.20	0.033	0.121/0.370
	number of days	0.10 ± 0.03	3.17	0.003	
biomass index	intercept	87.78 ± 22.87	3.84	< 0.001	0.037/0.701
	number of days	–0.53 ± 0.20	–2.69	0.010

### Effects of ALAN on herbivory

**Fig. 4:**
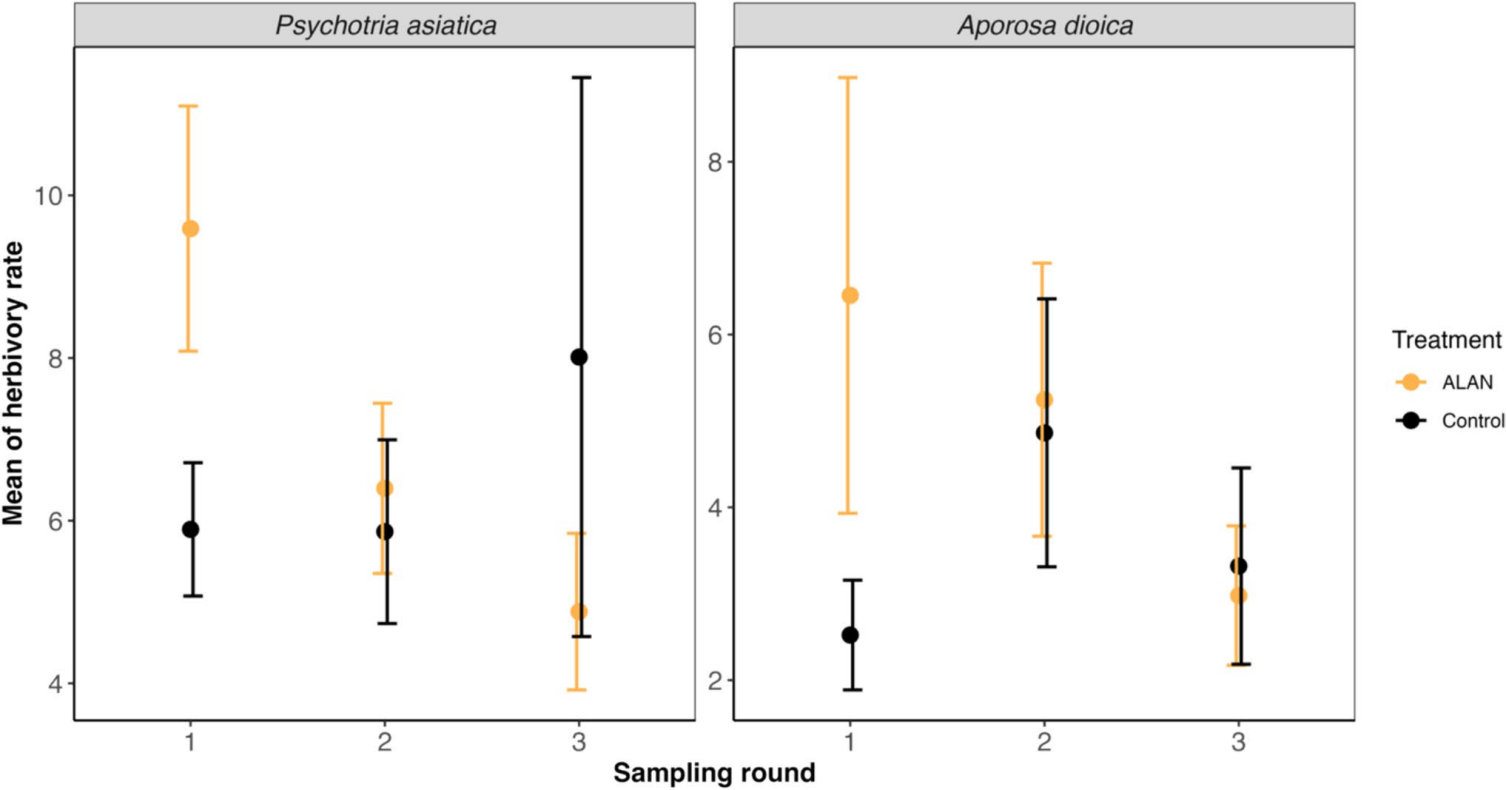
Mean herbivory rate of *P. asiatica* and *A. dioica* across light treatment and sampling round. Error bars indicate mean ± standard error. Number of days was categorized as sampling round based on which round of survey they belonged to for visualization. Surveys conducted between 29th April and 16th May 2024 were classified as the first round of sampling, surveys conducted between 29th May and 12th June 2024 were classified as the second round of sampling, otherwise as the third round

We conducted three rounds of surveys in all 14 plots and surveyed a total of 840 leaves (420 for each species). The mean (± s.d.) herbivory percentage of *P. asiatica* was 6.96 ± 3.61% on experiment plots and 6.59 ± 5.47% on control plots. The mean (± s.d.) herbivory percentage of *A. dioica* was 4.89 ± 4.71% on experiment plots and 3.57 ± 3.10% on control plots. The best-performing LMM (Table 2) suggests that light treatment, sampling round, and their combination all had significant effects (p = 0.045, p = 0.049, p = 0.044, respectively) on the percentage herbivory of *P. asiatica*. The percentage herbivory of *P. asiatica* was higher in illuminated plots at the beginning of the experiment but decreased as the experiment progressed. While the percentage herbivory of *P. asiatica* in control plots increased and was slightly higher than in the illuminated plot at the end (Fig. 4). Light treatment, sampling round and their combination did not show any significant effect on percentage herbivory of *A. dioica* (Table 2; Fig. 4). However, consistent with the results for *P. asiatica*, the herbivory rate of *A. dioica* in experimental plots was also higher in experimental plots in the early wet season and decreased over time (Fig. 4). We found no correlation between *N. pilipes* biomass and herbivory rates for *P. asiatica* (r = –0.25, p = 0.104) and *A. dioica* (r = –0.12, p = 0.452)*.*

**Table 2:** Parameter estimates (± s.e.), t-value, and P-value for each parameter included in the best-performing LMM examining the effect of introduction of ALAN and number of days on average herbivory percentage of *P. asiatica* and *A. dioica* at the 14 plots around Hong Kong. For each model, the marginal and conditional ${R}^{2}$ is presented to assess the contribution of random effect (location) to the sample variation. Note that no term in LMM for *A. dioica* is significant and the global LMM is demonstrated in the table

response variable	parameter	estimate ± s.e	t	p	marginal/conditional ${R}^{2}$
percentage (*P. asiatica*)	intercept	11.67 ± 2.51	4.66	< 0.001	0.102/0.217
	sampling round	–2.36 ± 1.13	–2.09	0.045	
	treatment (control)	–7.20 ± 3.44	–2.09	0.049	
	sampling round: treatment (control)	3.41 ± 1.59	2.15	0.044	
percentage (*A. dioica*)	intercept	8.37 ± 2.28	3.68	< 0.001	0.090/0.143
	sampling round	–1.74 ± 1.05	–1.65	0.107	
	treatment (control)	–5.60 ± 3.12	–1.79	0.089	
	sampling round: treatment (control)	2.14 ± 1.45	1.48	0.156

## Discussion

We found that ALAN (in our case, LED) did not alter *N. pilipes* growth or abundance but did result in elevated herbivory in the early wet season. This finding was counter to our prediction that ALAN would decrease the growth and abundance of the spider species and that cascading impacts on herbivory would be a consequence of this. Instead, herbivory rates appear sensitive to ALAN and increase in early spring but do not vary consistently in response to changes in spider biomass. The changes in herbivory in response to ALAN, independent of predator activity in the plots, highlight the complexity of artificial light impacts on species interactions, particularly in diverse tropical ecosystems.

We found that the herbivory rate of *P. asiatica* increased in the illuminated site and this effect was clearest in the early wet season (Fig. 4; Table 2). Although there is no clear evidence showing that ALAN affects the herbivory of *A. dioica*, its herbivory in illuminated plots also exhibited a similar pattern as in *P. asiatica*. The increase in herbivory in the early wet season could be due to an elevation in herbivore abundance and/or the prolonged time herbivores spend feeding due to ALAN. Consistent with our results, McMunn et al. (2019) found an increase in herbivory and local arthropod abundance when ALAN was introduced to the temperate grassland ecosystem.

We also observed a significant interaction between time and the ALAN effect (Fig. 4) – herbivory in ALAN plots decreased with time in both plant species. This decline of herbivory could be explained by changes in the maturity of leaves. As most herbivory of tropical leaves occurs when leaves are young and expanding (Coley and Barone 1996) and ALAN can accelerate leaf development (Czaja and Kołton 2022), the early maturation of leaves induced by ALAN may lead to a decrease in palatability and herbivory in both plant species. We may also have observed what is largely an herbivore effect, if ALAN attracted more arthropods to the lit plots but herbivory differences were only detectable during the early wet season when leaves are young and most palatable (but note that we detected small increases in herbivory for the focal plant species over time in the unlit plots). The impact of ALAN on other non-spider predators, especially birds, may also explain the interaction between herbivory and time. Previous studies have indicated that ALAN prolongs the foraging period of birds (Stracey et al. 2014; Russ et al. 2015). This elevated nocturnal activity of birds may result in increased predation on herbivores, consequently leading to a cascading effect on herbivory rates as the study progressed.

Our experimental results suggest that the growth and abundance of *N. pilipes* were not affected by the introduction of ALAN (Fig. 3; Table 1). This result is inconsistent with the prediction based on the previous study which indicates that the interception rate of prey decreases under ALAN (Yuen and Bonebrake 2017). Temporal differences could explain this discrepancy in spider predation between our result and the previous study. In Yuen and Bonebrake (2017), each selected *N. pilipes* was only sampled for one night for prey-interception rate, while we conducted semi-monthly sampling surveys to monitor the growth and abundance of the spider species. As such, the results in this study better integrate ALAN impacts over time and provide a more realistic estimate of ALAN impacts on the spider species. Alternatively, the positive and negative effects of ALAN on *N. pilipes* may offset each other. While exposure to ALAN can decrease the prey-interception rate and increase the mortality rates of juvenile orb-web spiders (Willmott et al. 2018), ALAN could potentially also cause aggregations of orb-weaver spiders—some spiders species are reported to construct webs near lights to utilize the phototaxis of prey (Heiling 1999), and phototaxis of prey could operate on a longer time scale, leading to an increase in the attraction rate of prey. These two effects may result in an overall equalising impact of ALAN on spider abundance and growth. Untangling such mechanisms, particularly for key mesopredators like spiders, should be a priority for future ALAN studies.

Light properties may also complicate the ecological effect of ALAN, as even low-intensity ALAN can induce behaviour or physiological differences in nocturnal species (Gaston et al. 2012). However, the spectral variation of ALAN can induce complexity in organism behaviour and species interaction. As major taxonomic groups have different spectral sensitivity, the broad-spectrum lamps adopted in our study created a disparity in visual perception among various taxonomic groups and induced a likely imbalance in species interactions (Davies et al. 2013). In our system, moths are a dominant food source for *N. pilipes* (Fan et al. 2009). Moths are more attracted to illumination types emitting UV than LED lights (Wakefield et al. 2016), potentially due to their peak spectral sensitivity in the UV. Since we used LED illumination in our experiment, this may not have cause sufficient change in the abundance of moths in the lit plots, thereby not inducing observable responses across trophic levels (specifically in the herbivory rate of *A. dioica* and biomass of *Nephila*). However, our light treatment resulted in elevated levels of herbivory in *P. asiatica*, which suggests that phototaxis of herbivores did occur in our experiment, but perhaps to a limited extent. Additionally, the spectral property of LED could have disrupted the relationship between *Nephila* and moths. Some moths use the UV-reflective markings on flowers as one of the visual cues to access nectar (Raguso and Willis 2005), and the UV-reflective patterns of *N. pilipes* generate a similar visual signal which increases the foraging success of spiders by luring pollinating moths (Tso et al. 2004). As the LED used in our study does not emit UV, it generates a UV-poor environment compared to ALAN-naïve habitats (MacGregor et al. 2015). This UV-poor environment could undermine the UV-reflective visual lures of *N. pilipes* and lead to lower predation success, complicating the effect of ALAN on *N. pilipes* growth rate.

Although we found no significant effect of ALAN on *N. pilipes*, this result is only based on the observation at the scale of a few metres around the light source. However, the effect of ALAN may operate at larger spatial scales (Kehoe et al. 2022). ALAN affects the predatory activities of other common predators such as bats and birds on larger spatial scales (Mariton et al. 2022), and these changes could negate any decreases in spider abundance in response to ALAN. However, our study did not exclude the predation of bats and birds on *N. pilipes* and the herbivore community.

The lack of any expected connection between increased herbivory and decreased spider growth and abundance in our results could be a consequence of multiple processes. As we did not observe the predicted response of abundance and growth of *N. pilipes* to ALAN, it is possible that the expected cascading effect on herbivory had no opportunity to occur at all. Additionally, there is a substantial knowledge gap of plant–herbivore interactions in pantropical regions (Slade and Ong 2023), and in our experiment, the herbivores of *A. dioica* were not observed in field (nor do we have clear records in the literature). In sum, more complex effects of ALAN on *N. pilipes* could be responsible for the lack of any detected predator–prey relationship while weak trophic relationships between *N. pilipes* and *A. dioica*/*P. asiatica* could also explain the lack of connection. Nevertheless, we encourage further investigation of ALAN impacts on predatory-prey relationships alongside changes in herbivory, while additional research on specific or isolated components could better clarify underlying mechanisms to explain effects (or lack of effects) of ALAN on species interactions.

Moths—the principal herbivores of the focal plant species and the primary prey of the orb-web spider—were not quantified in this study, constraining our ability to attribute the absence of an ALAN effect on spider biomass to changes in prey availability. However, our objective was not to resolve trophic links at the species level. Instead, we tested whether the reduction in nocturnal interception rates of prey in *N. pilipes* webs previously documented under ALAN (Yuen & Bonebrake 2017) induces detectable changes in herbivory and spider population parameters. Consequently, the study was designed to monitor ecological processes and importance of key mesopredators of subtropical forest ecosystems. Nevertheless, further investigation of the ALAN impacts on each of the trophic links in these ecosystems (plant growth, herbivory, predation, parasitism etc.) would better clarify the full scope of impacts of artificial light.

Tropical ecosystems host the greatest concentrations of biodiversity on the planet, but tropical regions are experiencing a rapid invasion of ALAN caused by the increasing human population and urbanisation (Secondi et al. 2020). Tropical ecosystems could be particularly vulnerable to ALAN, as the effect of light pollution is further amplified by heavy cloud cover of intertropical zones (Kyba et al. 2011; Secondi et al. 2020). Also, tropical species may be more sensitive to photoperiod change as the day length is more stable annually in the tropics than in temperate regions (Gaston et al. 2021). However, few studies have been conducted to assess ALAN’s effect on tropical biodiversity due to the complexity of tropical ecosystems per se and the insufficient mechanistic understanding of ALAN’s effect on ecosystems (Grubisic and van Grunsven 2021; Sanders et al. 2021). Our findings emphasise the potential sensitivity of herbivory and the resilience of *N. pilipes* under ALAN but also highlight the complexities of different species interactions in driving diverse fitness outcomes for species in subtropical forests, increasingly under the threat of urbanisation and associated anthropogenic impacts.

## Data Availability

The data and code that support the findings of this study are openly available in Figshare at [10.6084/m9.figshare.27273918.v1).

## Declarations

### Permits

For permits, we received Committee on the Use of Live Animals in Teaching and Research number 24–106 for this work.
